# Global burden and projections of cervical cancer attributable to unsafe sex and smoking, 1990–2034

**DOI:** 10.1371/journal.pone.0339923

**Published:** 2026-01-09

**Authors:** Jie Hu, Yajie Wang, Yan Liu, Zhengrong Cai

**Affiliations:** 1 Xiangya School of Public Health, Central South University, Changsha, Hunan, China; 2 School of Public Health, Hengyang Medical School, University of South China, Hengyang, Hunan, China; 3 Clinical Nursing Teaching and Research Section, The Second Xiangya Hospital, Central South University, Changsha, Hunan, China; Cedars-Sinai Heart Institute, UNITED STATES OF AMERICA

## Abstract

**Objectives:**

To analyze global trends and attributable risks (smoking and unsafe sex) in disease burden of cervical cancer from 1990 to 2034, considering different sociodemographic index (SDI) levels.

**Methods:**

Data from the Global Burden of Disease 2019 study was utilized. Quantile regression, restricted cubic spline, and Nordpred models were applied to analyze the relationship between cervical cancer age-standardized mortality rates (ASMRs), age-standardized Disability-Adjusted Life Years rates (ASDRs) and SDI, and predicted future trends.

**Results:**

From 1990 to 2019, global cervical cancer ASMR declined but the total deaths increased. Unsafe sex accounted for the highest disease burden, particularly in low-SDI regions, followed by smoking. By 2034, ASMR attributed to unsafe sex and smoking is projected to further decrease globally, but an upward trend is expected in specific regions including India (unsafe sex), China (smoking) and Russian Federation (unsafe sex and smoking).

**Conclusion:**

Unsafe sex is the leading risk factor for cervical cancer. Targeted strategies are needed for distinct age groups: enhanced prevention and screening for those aged 55–59 years, and optimized care for adults aged over 95 years. The key interventions, including HPV vaccination, screening, and smoking cessation programs, remain critical in low and low-middle SDI areas.

## 1. Introduction

Cervical cancer ranks as the fourth most prevalent cancer in women, following breast, colorectal, and lung cancers. It is unique among gynecological malignancies, as its etiology is well-defined, making it both preventable and treatable. Effective preventive strategies are already in place, including vaccination, screening, early diagnosis, and timely treatment [1]. However, despite global, regional and national efforts to eradicate cervical cancer, it remains a major health problem in the world, and poses a great threat to women’s health. The results of the GLOBOCAN2020 database show that there will be 604,000 new cases of cervical cancer and 342,000 deaths around the world in 2020, accounting for 6.5% of new cases and 7.7% of deaths among females, with a crude incidence rate of cervical cancer of 15.6 per 100,000 and a crude mortality rate of 8.8 per 100,000 globally [2,3].

Cervical cancer leads the list of female tumors in terms of morbidity and mortality in many countries, but the mortality rate varies widely and the pattern of incidence is highly heterogeneous, with significant differences between countries and regions [4]. Eastern Africa, Southern Africa, Central Africa, Melanesia and Western Africa experience the highest incidence rates, while Western Europe, Northern Africa, North America, Australia, and Western Asia have the lowest age-standardized incidence rates. One of the reasons for the heterogeneity of the disease pattern is socio-economic gradient. Studies have indicated that there is about 90% of cervical cancer cases occurring in low- and middle-income countries, where the mortality rate is 18 times higher than in rich countries [5]. Similarly, the incidence of cervical cancer shows a significant increase in countries with a low Human Development Index (HDI). Morbidity and mortality in low HDI countries is three and six times higher than that in high HDI countries, respectively [2]. The peak incidence of cervical cancer globally is 50–54 years of age, around 40 years in developed countries and 55–69 years in developing countries [4,6].

The main causative factor of cervical cancer is human papillomavirus (HPV), which is sexually transmitted. Persistent infection with high-risk HPV is essential for cervical cancer and its precancerous lesions [7,8]. There are 100 known subtypes of HPV, 12 of which are high-risk, and the main subtypes that cause 70% of cervical cancers are HPV 16 and 18. Factors such as smoking, early age of sexual debut, oral hormonal contraceptives, multiple sexual partners and unprotected sex increase the risk of HPV infection, which contributes to the disease burden of cervical cancer [9].

At present, the prevention and control of cervical cancer are mainly primary and secondary prevention. Primary prevention is HPV vaccination and health education, and secondary prevention is cervical cancer screening as well as early diagnosis, and tertiary prevention is clinical treatment to improve life quality of patients [10,11]. The HPV vaccine is an effective preventive measure against cervical cancer [12]. HPV vaccination can lead to a lower rate of HPV infection and effectively prevent most cervical cancers and genital warts [13].

Although previous studies have explored the impact of sociodemographic index (SDI) on the burden of disease in cervical cancer, they have not explicitly clarified the association under different risk factors. Furthermore, there is limited studies exploring the differences of this association at various levels of SDI. Therefore, this paper incorporates worldwide data on cervical cancer and utilizes various statistical analysis methods, focusing on studying the burden of attributable risk of cervical cancer at different levels of SDI globally and reasonably predicting the trend of attributable mortality of cervical cancer globally in the next 15 years.

## 2. Method

### 2.1 Data sources

Data was obtained from the official website of the Global Burden of Disease Study 2019 (GBD2019) (http://ghdx.healthdata.org/gbd-results-tool), the study was led by the Institute for Health Metrics and Evaluation (IHME) at the University of Washington. GBD2019 utilizes baseline datasets to quantify health levels and trends, and provides comprehensive estimates of the burden for 369 injuries, diseases and 87 risk factors in 204 countries and territories around the world [14–16], making it the largest and most detailed global burden of disease study to date.

The ICD-10 codes for Cervical Cancer are C53, C53.0, C53.1, C53.8, C53.9 according to the Classification of Diseases codes. Risk factors associated with cervical cancer include smoking and unsafe sex [14,17]. Smoking was defined based on GBD 2019 criteria, including current smokers (daily or occasional use of smoked tobacco products) and former smokers (cessation for ≥6 months) [14,18]. Exposure was quantified using standard indicators (e.g., pack-years for current smokers and years since cessation for former smokers) [14]. These data sources of smoking were cross-sectional nationally representative household surveys, and primary data was extracted from individual level microdata and survey report tabulations. Unsafe sex is defined as the risk of disease due to sexual transmission, and the theoretical minimum level for unsafe sex was defined as the absence of disease transmission through sexual contact [19]. The primary data sources of unsafe sex were UNAIDS, the European CDC, and the US CDC. Age-standardized mortality rate (ASMR) and age-standardized Disability-Adjusted Life Years rate (ASDR) are standardized according to the GBD2019 World Standard Population Composition. Age-specific mortality rate (ASpMR) and age-specific incidence rate (ASpIR) are mortality rate and incidence rate estimated by age, respectively.

Disability-adjusted life years (DALYs), which represent the time lost due to disability or death caused by a specific disease, are considered an appropriate indicator to quantify the burden of disease and injury, cost-effectiveness and resource allocation [20]. It is a combination of Years of Life Lost (YLL) and Years of Life with disability (YLD). YLL is the number of years of life lost due to premature death below life expectancy caused by a disease [21]. YLD is the number of years of healthy life lost as a result of disability caused by a disease, it was calculated by a standardized method using prevalence of each disease sequelae and assigned disability weights (DW) [22]. GBD2019 used comparative risk assessment (CRA) to estimate the proportion of deaths and DALYs attributable to risk factors by comparing the actual exposure of the target population with the Theoretical Minimum Risk Exposure Level (TMREL), holding other risk factors constant. In addition, population attributable fractions (PAFs) corresponding to age-sex-region-year were estimated for each risk factor to calculate the percentage of morbidity or mortality from a disease attributable to exposure to a particular risk factor out of the total morbidity or mortality from that disease in the total population [14].

In the 2015 Global Burden of Disease study (GBD 2015), a Socio-demographic Index (SDI) similar to the Human Development Index was computed for the first time [23]. Developed by GBD researchers, SDI is a composite indicator of development status strongly correlated with health outcomes [15], which consists of three components: average education in individuals aged ≥ 15 years, lag distributed income (LDI) per capita and the total fertility rate of the population aged < 25 years. The value of SDI ranges from 0 to 1. 204 countries and territories were divided into 5 categories based on SDI, including low (0–0.454743), low-middle (0.454743–0.607679), middle (0.607679–0.689504), high-middle (0.689504–0.805129), and high (0.805129–1) [14].

### 2.2 Statistical analysis

#### 2.2.1 ASR and EAPC.

We used age-standardized rates (ASRs) to analyze trends in the burden of cervical cancer over the past 30 years, where age-standardized mortality rates (ASMRs) and age-standardized DALY rates (ASDRs) were calculated as follows [24]:


$$ASR=\frac{\sum_{\text{i}=\text{1}}^{A}a_{i}w_{i}}{\sum_{i=\text{1}}^{A}w_{i}}\times \text{1}\text{0}\text{0}\text{0}\text{0}\text{0}$$

In the i-th age subgroup, a_i_ denotes the age-specific mortality or DALY rate, and w_i_ represents the number (or weight) of people in the selected standard population.

Estimated annual percentage change (EAPC) was adopted to accurately assess the epidemiological trend of cervical cancer mortality rate and disability adjusted life age.

EAPC and its 95% uncertainty interval (UI) were calculated by a linear regression model as follows, where x refers to the calendar year:


ln(ASR)=α+βx+ϵ

$$\text{EAPC}=\text{100}\times (e^{\beta }-\text{1})$$

If the upper limit and confidence interval of the EAPC are less than zero, the ASR represents the mortality or the disability adjusted life year of cervical cancer on the decline, and vice versa [25].

#### 2.2.2 Quantile regression.

Quantile regression is a linear regression under different quantile conditions, which is based on the principle of weighted least squares method. It is a class of regression models rather than a single model [26]. Quantile regression can fit multiple regression lines based on quantile points, providing more comprehensive information to help understand the full picture of the conditional distribution of the dependent variable, and has better robustness to outliers [27].

#### 2.2.3 Restricted cubic spline (RCS).

RCS is one of the most common methods for analyzing nonlinear relationships. It is a powerful and flexible nonparametric method that does not require prior specification of the functional form, and avoids overfitting while maintaining curve smoothness. RCS is essentially a regression spline with additional requirements. A regression spline is a segmented polynomial, which is generally required to be continuous and second-order derivable at each segmentation point in order to ensure the smoothness of the curve [28]. These segmentation points are called “nodes” and the number of nodes affects the fit of the model. The RCS models were optimized for accuracy and overfitting using the Akaike information criterion (AIC). The knots were determined based on the minimum AIC value. The recommended number of nodes is 3–5, with 3 chosen because it minimized AIC compared to models with 4 or 5 knots.

#### 2.2.4 Nordpred age-period-cohort.

The prediction of chronic disease incidence and death is complicated by the influence of factors such as age, period, and birth cohort. The Nordpred age-period cohort model predicts based on the independent effects of age, period, and cohort, using different trend parameters for prediction and emphasizing the weighting of recent trends. It overcomes the problem of exponential growth of rates over time in the classical APC model and makes the prediction results more accurate [29]. At the same time, this model has been shown to be a better predictor of estimated tumor incidence and mortality trends, and has been widely used in studies in different countries [30,31]. The Nordpred model makes predictions based on either a power function or a Poisson distribution function, where power function is more suitable for cancer incidence and mortality projection. We have divided 1990–2019 into six five-year intervals (1990–1994, 1995–1999, 2000–2004, 2005–2009, 2010–2014, and 2015–2019) to predict the trends of cervical cancer mortality from 2020 to 2034. We selected five countries (the United States of America, the Russian Federation, China, India, and Pakistan) by systematically choosing the most populous country within each SDI quintile, ensuring that each selected country represents a different SDI level. The R software 4.5.2 was applied for statistical analyses, and P <  0.05 considered statistically significant.

## 3. Results

### 3.1 Temporal trends in different SDI levels

In Table 1, we showed the number of cervical cancer deaths, ASMR, and EAPC globally, in the five SDI classes, and in 21 regions from 1990 to 2019. The number of deaths is increasing at a global level, with 184,527 (95% UI: 164,836−218,942) deaths in 1990 and 280,479 (95% UI: 238,864−313,930) deaths in 2019, representing a 52% growth of the total deaths rate of this disease. The ASMR showed a downward trend, with an average annual decrease of 0.93% (EAPC = −0.93; 95% UI: −0.98 - −0.88), from 8.48/100000 people (95% UI, 7.59–10.07) in 1990 to 6.51/100000 people (95% UI, 5.55–7.29) in 2019. For specific DALYs and ASDR in 1990 and 2019, please refer to Table 2. The overall DALY count is on an upward trend, growing from 6,176,248 DALY (95% UI: 5437672−7316926) in 1990–8,955,013 DALY (95% UI: 7547733−9978462) in 2019, with total DALY rate growth of 44.99%. The ASDR decrease obviously, with an average annual decrease of 0.95% (EAPC = −0.95, 95% UI: −1.00 - −0.90).

**Table 1:** Number of deaths and age-standardized death rates for cervical cancer globally, in SDI regions and geographic areas, 1990 and 2019, and percentage change in EAPC from 1990 to 2019.

Characteristics	1990		2019		1990-2019
	Deaths No. × 10^3^ (95%UI)	ASMR per 100,000 No. (95%UI)	Deaths No. × 10^3^ (95%UI)	ASMR per 100,000 No. (95%UI)	EAPC in ASMR No. (95%UI)
Global	184.53 (164.84-218.94)	8.48 (7.59-10.07)	280.48 (238.86-313.93)	6.51 (5.55-7.29)	−0.93 (−0.98--0.88)
**Sex**					
Female	184.53 (164.84-218.94)	8.48 (7.59-10.07)	280.48 (238.86-313.93)	6.51 (5.55-7.29)	−0.93 (−0.98--0.88)
**SDI region**					
High SDI	25.22 (23.28-26.19)	4.56 (4.22-4.71)	26.17 (22.82-28.15)	2.90 (2.60-3.10)	−1.57 (−1.68--1.46)
High-middle SDI	41.35 (38.69-48.40)	6.95 (6.50-8.13)	51.77 (41.66-57.87)	4.89 (3.92-5.47)	−1.25 (−1.31--1.19)
Middle SDI	52.53 (46.63-65.12)	9.32 (8.31-11.54)	90.10 (71.33-103.20)	6.78 (5.40-7.76)	−1.03 (−1.09--0.97)
Low-middle SDI	39.21 (32.46-50.05)	11.71 (9.73-15.05)	66.68 (57.27-81.24)	8.85 (7.62-10.83)	−1.04 (−1.12--0.96)
Low SDI	26.08 (20.23-32.11)	19.18 (15.00-23.66)	45.54 (35.80-56.26)	15.05 (11.92-18.46)	−0.90 (−0.94--0.87)
**GBD region**					
Andean Latin America	2.33 (1.96-2.76)	20.39 (17.22-24.05)	4.28 (3.32-5.38)	14.37 (11.18-18.04)	−1.33 (−1.44--1.22)
Australasia	0.45 (0.39-0.48)	3.73 (3.16-3.92)	0.52 (0.45-0.58)	2.17 (1.88-2.40)	−1.57 (−1.95--1.19)
Caribbean	2.23 (1.75-2.55)	15.83 (12.55-18.04)	3.47 (2.72-4.26)	12.95 (10.11-15.96)	−0.65 (−0.72--0.58)
Central Asia	2.72 (2.48-2.89)	9.81 (8.95-10.43)	3.42 (3.00-3.93)	7.58 (6.68-8.70)	−0.75 (−0.88--0.62)
Central Europe	8.00 (7.60-8.53)	10.14 (9.63-10.79)	6.88 (5.82-7.99)	6.65 (5.59-7.75)	−1.57 (−1.68--1.47)
Central Latin America	9.59 (8.70-10.01)	20.35 (18.19-21.28)	13.83 (11.53-16.80)	10.65 (8.91-12.92)	−2.61 (−2.76--2.46)
Central Sub-Saharan Africa	3.72 (2.61-4.84)	26.27 (18.78-34.25)	7.30 (4.91-10.06)	21.67 (14.49-30.24)	−0.67 (−0.78--0.55)
East Asia	28.40 (22.32-46.14)	6.05 (4.77-9.76)	55.96 (33.19-71.36)	5.18 (3.09-6.59)	−0.05 (−0.28-0.18)
Eastern Europe	12.94 (10.95-13.86)	7.62 (6.50-8.19)	10.04 (8.47-11.91)	5.54 (4.62-6.61)	−1.38 (−1.54--1.21)
Eastern Sub-Saharan Africa	11.94 (9.08-14.92)	26.51 (20.07-33.45)	21.11 (15.48-27.86)	21.13 (15.15-27.62)	−0.90 (−0.96--0.84)
High-income Asia Pacific	4.64 (4.35-5.32)	4.20 (3.93-4.81)	5.60 (4.58-6.22)	2.70 (2.22-2.96)	−1.52 (−1.59--1.44)
High-income North America	6.74 (5.97-7.04)	3.71 (3.25-3.86)	8.80 (7.47-9.34)	2.99 (2.55-3.15)	−0.69 (−0.83--0.55)
North Africa and Middle East	3.97 (2.82-4.53)	4.37 (3.10-4.99)	7.00 (5.44-8.31)	3.15 (2.47-3.69)	−1.11 (−1.21--1.01)
Oceania	0.30 (0.22-0.42)	18.16 (13.38-25.22)	0.67 (0.45-0.91)	16.41 (11.50-22.19)	−0.18 (−0.27--0.08)
South Asia	33.33 (26.22-39.95)	10.49 (8.29-12.62)	53.30 (42.87-69.95)	7.01 (5.66-9.21)	−1.60 (−1.77--1.42)
Southeast Asia	16.71 (12.57-21.47)	11.05 (8.36-14.54)	25.13 (20.52-34.98)	7.36 (6.03-10.33)	−1.52 (−1.61--1.44)
Southern Latin America	3.07 (2.89-3.31)	12.34 (11.62-13.30)	4.18 (3.55-4.60)	9.64 (8.17-10.56)	−1.01 (−1.12--0.89)
Southern Sub-Saharan Africa	3.24 (2.48-4.07)	19.17 (14.63-24.27)	6.56 (5.39-7.75)	19.34 (15.82-22.77)	0.46 (0.19-0.72)
Tropical Latin America	7.69 (7.21-8.88)	14.72 (13.70-17.12)	11.58 (10.72-13.66)	8.69 (8.04-10.23)	−2.01 (−2.11--1.92)
Western Europe	13.13 (12.04-13.61)	4.36 (3.99-4.51)	11.75 (10.27-12.69)	2.65 (2.38-2.85)	−1.65 (−1.75--1.54)
Western Sub-Saharan Africa	9.38 (7.56-12.31)	19.74 (15.94-25.76)	19.09 (15.04-24.01)	16.83 (13.38-21.00)	−0.48 (−0.53--0.43)

**Table 2:** Number of DALY and age-standardized DALY rates for cervical cancer globally, in SDI regions and geographic areas, 1990 and 2019, and percentage change in EAPC from 1990 to 2019.

Characteristics	1990		2019		1990-2019
	DALY No. × 10^3^ (95%UI)	ASDR per 100,000 No. (95%UI)	DALY No. × 10^3^ (95%UI)	ASDR per 100,000 No. (95%UI)	EAPC in ASDR No. (95%UI)
Global	6176.25 (5437.67-7316.93)	275.05 (242.75-326.15)	8955.01 (7547.73-9978.46)	210.64 (177.67-234.85)	−0.95 (−1.00--0.90)
**Sex**					
Female	6176.25 (5437.67-7316.93)	275.05 (242.75-326.15)	8955.01 (7547.73-9978.46)	210.64 (177.67-234.85)	−0.95 (−1.00--0.90)
**SDI region**					
High SDI	725.85 (665.20-752.59)	143.23 (130.30-148.45)	672.11 (608.75-722.00)	89.72 (81.88-95.85)	−1.62 (−1.74--1.49)
High-middle SDI	1274.57 (1193.74-1497.01)	215.21 (201.46-252.85)	1543.70 (1236.00-1729.87)	154.69 (124.02-173.51)	−1.16 (−1.22--1.10)
Middle SDI	1790.63 (1588.48-2223.17)	287.82 (255.02-356.33)	2817.25 (2223.19-3217.72)	204.60 (161.92-233.49)	−1.11 (−1.17--1.05)
Low-middle SDI	1419.29 (1160.75-1789.13)	381.90 (315.26-485.47)	2282.24 (1948.33-2722.93)	285.64 (244.64-342.16)	−1.08 (−1.17--0.99)
Low SDI	961.20 (732.59-1179.64)	630.59 (487.61-777.41)	1632.49 (1271.61-2044.29)	477.53 (374.33-591.38)	−1.05 (−1.09--1.00)
**GBD region**					
Andean Latin America	79.25 (66.33-92.90)	633.25 (529.46-743.28)	129.59 (99.42-165.40)	422.28 (323.97-538.40)	−1.54 (−1.66--1.42)
Australasia	13.93 (11.33-14.68)	120.58 (96.79-127.24)	13.58 (11.84-15.06)	65.47 (57.37-72.70)	−1.83 (−2.26--1.41)
Caribbean	77.36 (58.62-90.62)	526.18 (401.54-612.81)	114.71 (86.80-145.02)	438.19 (328.34-557.85)	−0.63 (−0.69--0.56)
Central Asia	89.50 (83.58-95.46)	317.62 (299.06-339.68)	119.72 (103.95-138.54)	249.41 (217.42-288.15)	−0.72 (−0.87--0.58)
Central Europe	251.14 (236.32-264.12)	333.96 (312.61-350.79)	190.26 (159.63-221.33)	212.08 (177.26-247.37)	−1.73 (−1.86--1.60)
Central Latin America	323.66 (302.50-337.95)	610.29 (562.25-636.93)	436.92 (361.75-538.52)	328.59 (272.54-404.33)	−2.48 (−2.63--2.32)
Central Sub-Saharan Africa	135.73 (92.58-179.24)	848.19 (592.20-1109.46)	261.63 (176.04-360.43)	678.72 (454.78-932.08)	−0.77 (−0.90--0.65)
East Asia	921.11 (714.66-1517.19)	182.74 (142.14-297.89)	1696.32 (972.55-2166.63)	159.12 (91.62-202.95)	0.02 (−0.20-0.24)
Eastern Europe	368.58 (319.59-396.93)	233.88 (203.92-253.70)	308.61 (255.79-369.57)	192.88 (156.89-231.51)	−0.89 (−1.09--0.69)
Eastern Sub-Saharan Africa	444.59 (335.91-562.71)	876.65 (666.35-1103.42)	758.61 (557.09-1022.32)	660.28 (484.65-874.17)	−1.13 (−1.21--1.06)
High-income Asia Pacific	136.98 (127.56-156.31)	126.77 (118.36-144.52)	133.64 (109.97-146.61)	85.67 (67.59-93.64)	−1.29 (−1.35--1.23)
High-income North America	206.88 (177.72.1-216.04)	123.61 (105.30-129.07)	245.96 (211.94-259.26)	96.55 (83.94-101.88)	−0.84 (−0.99--0.69)
North Africa and Middle East	133.00 (94.24-152.10)	130.16 (92.26-148.26)	221.93 (169.20-268.19)	88.28 (68.42-105.74)	−1.37 (−1.44--1.29)
Oceania	11.34 (7.93-15.07)	579.78 (417.98-793.65)	24.91 (16.09-34.06)	521.37 (347.49-709.69)	−0.17 (−0.27--0.08)
South Asia	1230.95 (969.02-1468.76)	345.39 (271.36-413.30)	1833.69 (1466.66-2370.91)	226.59 (181.92-292.64)	−1.64 (−1.83--1.45)
Southeast Asia	592.31 (440.72-733.90)	354.96 (266.86-447.24)	808.25 (653.21-1088.29)	223.36 (181.44-302.65)	−1.73 (−1.83--1.64)
Southern Latin America	101.76 (96.27-107.78)	413.34 (390.66-437.19)	127.49 (105.40-140.05)	317.23 (260.30-348.08)	−1.07 (−1.18--0.96)
Southern Sub-Saharan Africa	116.35 (89.85-142.79)	633.63 (488.91-782.66)	213.94 (173.97-254.95)	586.79 (476.20-698.37)	0.23 (−0.04-0.51)
Tropical Latin America	262.80 (247.91-303.91)	455.15 (428.99-524.50)	365.28 (340.28-419.75)	274.27 (255.50-314.34)	−1.95 (−2.05--1.85)
Western Europe	351.50 (321.56-363.85)	134.35 (119.97-138.99)	277.36 (248.48-299.74)	79.19 (71.88-85.34)	−1.77 (−1.90--1.64)
Western Sub-Saharan Africa	327.53 (261.59-424.73)	626.47 (502.32-815.10)	672.60 (524.74-854.86)	507.97 (398.99-640.76)	−0.69 (−0.74--0.64)

**Fig 1:**
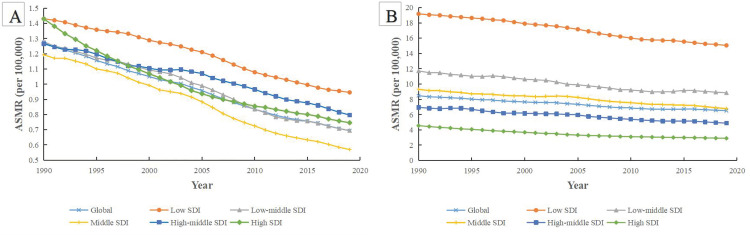
The attributable ASMR of cervical cancer at different SDI quantile from 1990 to 2019. **(A)** The ASMR of cervical cancer attributable to smoking at different SDI quantile from 1990 to 2019. **(B)** The ASMR of cervical cancer attributable to unsafe sex at different SDI quantile from 1990 to 2019. ASMR: age-standardized mortality rate; SDI: sociodemographic index.

Fig 1A and 1B presented the ASMRs trends of cervical cancer attributable to smoking and unsafe sex at five different SDI levels from 1990 to 2019, respectively. The ASMRs for cervical cancer attributable to both risk factors showed an overall decreasing trend around the world and in different SDI levels. The values of ASMRs attributed to unsafe sex were significantly higher than those of smoking. The ASMR are highest in low SDI areas, where the ASMR attributable to smoking decreased from 1.43/100,000 to 0.95/100,000 from 1990 to 2019, and the ASMR attributable to unsafe sex declined from 19.18/100,000 to 15.05/100,000. The greatest decreases in ASMR attributed to smoking were observed in the high SDI countries, which declined by 47.80% (from 1.43 to 0.75 per 100000 people). While the ASMR values attributed to unsafe sex decreased the most in low SDI countries, from 19.18 to 15.05 per 100000 people. The ASDRs for cervical cancer attributed to these two risk factors followed similar trends to those of ASMRs (S1 Fig).

### 3.2 The age-specific mortality and incidence rates by age group

**Fig 2:**
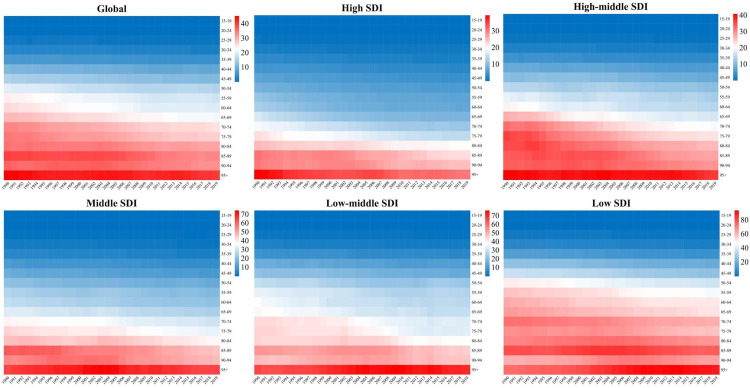
The age-specific mortality rates for cervical cancer attributable to unsafe sex by SDI, 1990-2019. SDI: socio-demographic index.

ASpMRs for cervical cancer attributable to unsafe sex and smoking from five different SDI levels globally are displayed in Figs 2 and S2. ASpMRs for cervical cancer attributable to both risk factors showed a downward trend across SDI levels, and ASpMRs attributable to unsafe sex were significantly higher than those of smoking. The age group with the highest mortality from cervical cancer associated with unsafe sex was predominantly over 95 years in five SDI levels. The highest mortality in the 95 + age group reflects challenges in managing advanced disease, emphasizing the need for optimized clinical care and palliative support, particularly in low SDI areas where mortality rose from 74.04 per 100000 people in 1990 to 89.00 per 100000 people in 2019. Between 1990 and 2019, the global ASpMR attributable to unsafe sex decreased from 45.50 per 100,000 to 40.45 per 100,000 (Fig 2), and the rate attributable to smoking showed a downtrend from 6.55 per 100,000 to 3.89 per 100,000 (S2 Fig).

We also collected data from five SDI levels in the world to investigate the ASpIRs of cervical cancer attributable to the two risk factors, and the rates are presented in S3 Fig. The incidence rates in 2019 were lower than those in 1990 in most age groups, and the incidence of 55–59-years was observed the highest. The highest incidence in 55–59 years highlights the need for targeted screening and prevention in this group to detect precancerous lesions early. The incidence of 55–59-years for this cancer showed the lowest in high SDI quantile, decreasing from 27.00 per 100000 in 1990 to 19.47 per 100000 in 2019. While the highest incidence of this age group from cervical cancer was observed in the low SDI level. The incidence was 79.06 per 100000 and 66.32 per 100000 in 1990 and 2019, respectively.

### 3.3 The proportion of YLL in DALY

As shown in S4 Fig, the component ratios of YLL for cervical cancer attributed to unsafe sex and smoking in DALYs were above 95% worldwide, and both were on a downward trend. The proportions of YLL attributed to unsafe sex was higher in high-middle SDI areas, while those of smoking was higher in other SDI quantiles. The percentage of YLL of cervical cancer attributed to both risk factors declined progressively with increasing SDI level.

### 3.4 The relationship between SDI and ASMR or ASDR of cervical cancer

**Fig 3:**
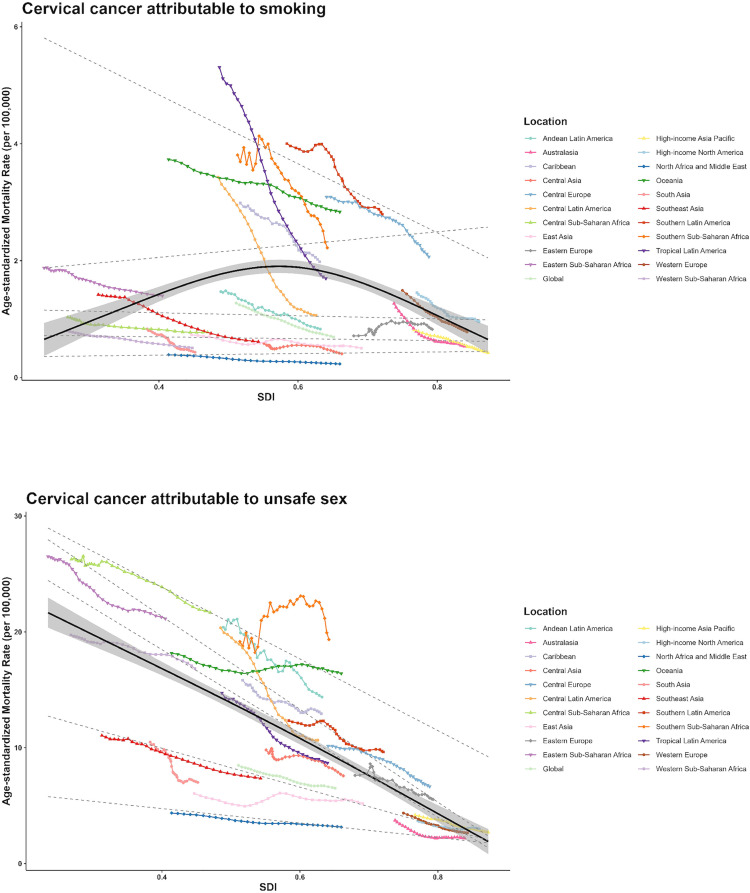
The relationship between the ASMR (per 100000) of cervical cancer attributable to smoking, unsafe sex and SDI from 1990 to 2019 in 21 regions of the world classified by GBD. The solid black line represents the fitted restricted cubic spline (RCS) curve. Each colored line represents the time trend in the designated and each point represents a specific year in the region. The gray dotted line represents the results of quantile regression, from top down to next are the results of P_95_, P_75_, P_50_, P_25_ and P_5_. ASMR: age-standardized mortality rate; SDI: socio-demographic index; GBD: Global Burden of Diseases; RCS: Restricted Cubic Spline.

We used RCS and quantile regression to analyze the relationship between ASMR, ASDR of cervical cancer and SDI values in 21 regions classified by GBD, which was presented in Figs 3 and S5, respectively. The results of RCS illustrate that when the SDI was close to 0.6, the ASMR of cervical cancer attributed to smoking was the highest. The findings of quantile regression illustrate how the impact of the SDI on ASMR of cervical cancer attributable to smoking and unsafe sex across different quantile levels. For ASMR of cervical cancer attributed to smoking, a statistically significant negative association was observed only at the second quantile (P_25_), while the association was not significant at other quantiles (P_5_, P_50_, P_75_, P_95_). The P_25_ curves indicated that the ASMR of cervical cancer attributed to smoking decreased with increasing SDI in those countries or regions where ASMR distributed around the 25th percentile (Fig 3). However, five quantiles were significant for ASMR of cervical cancer attributed to unsafe sex. The quantile regression showed that at higher ASMR quantiles, the decline in cervical cancer ASMR attributable to unsafe sex was steeper as SDI increased, indicating that regions with the highest burden experienced the largest reductions with rising socioeconomic development (Fig 3). A similar result was obtained for the relationship between ASDR and SDI (S5 Fig).

### 3.5 Predictions of ASMR for cervical cancer in different SDI countries from 2020 to 2034

**Fig 4:**
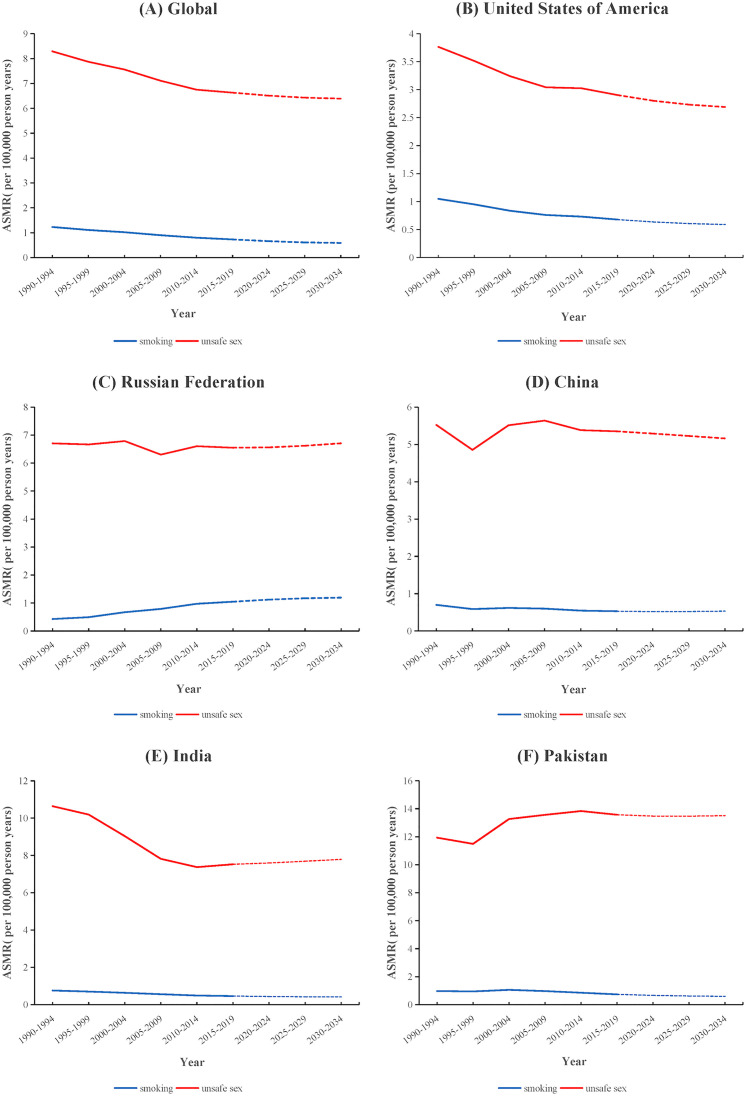
Predictions of ASMR of cervical cancer attributable to unsafe sex and smoking in different SDI countries from 2020 to 2034. ASMR: age-standardized mortality rate; SDI: sociodemographic index.

The projected ASMR trends for cervical cancer attributed to both two risk factors in five different SDI countries were exhibited in Fig 4. In the next 15 years, the ASMR for cervical cancer attributed to unsafe sex was predicted to decrease in global, United States of America, China and Pakistan, while that in Russian Federation and India were projected to increase to 6.71 per 100000 and 7.79 per 100000 in 2034, respectively. The ASMR for cervical cancer attributed to smoking was projected to show an upward trend to 0.53 per 100,000 in China and 1.19 per 100,000 in Russian Federation. In contrast, the values in other three countries and global were projected to decline.

## 4. Discussion

We observed a progressive reduction in the disease burden of cervical cancer from 1990 to 2019. Unsafe sex was a more significant contributor than smoking, consistent with previous studies on epidemiological trends and attributable risk burden. We also identified the age groups with the highest incidence and mortality rates: 55–59 years and over 95 years. Comparing our findings to older studies revealed that years of life lost (YLL) attributed to both risk factors accounted for over 95% of DALY and the proportion of years lived with disability (YLD) showed an upward trend. This likely stems from the fact that the disease burden of cervical cancer primarily arises from YLL due to premature mortality. Additionally early screening reduces the risk of premature death from cervical cancer, which in turn increases the burden of loss of healthy life expectancy because of disability.

This paper presents findings suggesting the burden of cervical cancer linked to smoking peaks at an SDI close to 0.6, corresponding to a Low-middle SDI. This finding aligns well with the ‘tobacco transition’ theory [32], which posits that as a country develops, the smoking rate among women tends to rise before public health infrastructure for tobacco control is fully established. This leads to a temporary peak in burden, but strong control measures eventually lead to a decline at very high SDI levels. In 2005, the World Health Organization promulgated the Framework Convention on Tobacco Control [33]. which was formally adopted for implementation in many countries and regions. However, the situation with regard to smoking-attributable diseases among women remains critical due to wide disparities in the implementation of tobacco control in different countries and regions, especially in low and low-middle SDI areas, where the issue of women’s tobacco use is not yet fully recognized [34]. Global cancer statistics 2018 showed that 290,000 of the 570,000 new cases of cervical cancer worldwide occur in women in low- and middle-income countries [35]. A study by Vaccarella et al [36] also exhibited that 86% of the cervical cancer burden in the world occurs in Africa, Latin America and the Caribbean and Asia, with a higher concentration of low SDI countries in these regions. Thus, smoking may be one of the reasons for the more serious burden of cervical cancer among women in low and low-middle SDI countries. There are several explanatory hypotheses about the association between smoking and cervical cancer, including a direct oncogenic effect on chemical carcinogenesis [37], the suppression of cell-mediated local immunity effect [38], the synergistic effect of HPV infection [39], or oxidative stress [40]. However, the exact role of smoking in cervical carcinogenesis is not known. Furthermore, it should be noted that passive smoking is associated with higher risk of cervical cancer incidence [41]. According to WHO report on the global tobacco epidemic 2021, approximately 5% of cervical cancer cases worldwide can be attributed to passive smoking. Proactive strategies for tobacco control to protect women health is warranted.

In addition, our study found that the burden of cervical cancer attributed to unsafe sex was highest in low SDI countries and lowest in high SDI countries. The HPV virus is the most important risk factor for cervical cancer, and the main route of infection is through unsafe sex. While the association between unsafe sex and HPV is well-established, boarder factors driving this behavior require further elaboration. First, socioeconomic disparities play a role. Marginalized populations in lower SDI regions with limited healthcare access are more likely to engage in unsafe sex due to barriers in resource availability [42]. Second, people are more likely to engage in unsafe sex because of limited access to comprehensive sexual health education. Research shows that the lack of sex education is significantly associated with risky sexual behaviors among teenagers, such as unprotected sex and multiple sexual partners [43]. Third, gender inequalities and power imbalances can constrain women’s ability to negotiate safer sex. A review investigated men’s influence on women’s reproductive health showed that the imbalance of gender power (such as male dominance in decision-making) may prevent women from demanding the use of condoms or refusing high-risk sexual behaviors [44]. Furthermore, stigma around sexual health discussions may hinder access to preventive resources like condoms or HPV vaccination [45]. Addressing these underlying factors is critical for designing targeted interventions to reduce HPV exposure, as they directly influence the adoption of safer sexual practices beyond individual behavioral choices.

Emerging evidence suggests potential interactions between smoking and unsafe sexual behaviors in influencing cervical cancer risk, which merits tentative consideration. Smoking may exacerbate the carcinogenic effects of HPV acquired through unsafe sexual behaviors by local immunologic changes associated with cigarette smoking and prolonging HPV persistence [38]. Epidemiological studies have also shown that HPV-positive smokers have a higher risk of developing cervical cancer and a more severe disease burden compared to non-smokers [46]. However, it is important to note that the ecological, population-level design of the previous study precludes formal testing or validation of a direct interaction between these two factors. In Low or Low-middle SDI regions with a high smoking rate and insufficient sexual health resources, the co-occurrence of these risk factors may contribute to elevated cervical cancer burden. Therefore, multi-sectoral policies could consider addressing structural barriers by expanding access to sexual health resources (e.g., condoms, HPV vaccination) alongside tobacco control measures (e.g., smoking cessation programs, smoke-free regulations) to reduce co-exposure to both risk factors. Further individual-level studies (e.g., cohort or case-control designs) are needed to elucidate whether and how these factors interact to influence cervical cancer risk.

Projections indicated that cervical cancer mortality attributable to both risk factors will decrease globally and in the United States over the next 15 years, consistent with the findings of Shujuan Lin et al [47]. However, our predictions diverge for certain countries: ASMR of cervical cancer attributable to smoking is projected to rise in China, while ASMR of cervical cancer attributable to unsafe sex is predicted to increase in India. These discrepancies may be partly explained by methodological differences— Lin’s study estimated overall cervical cancer mortality, whereas our analysis provides risk-factor-specific projections. In addition, country-specific epidemiological trends likely contribute to these upward patterns that opposite to the overall trend (e.g., increasing smoking prevalence among women in China [48] and the slower pace of HPV vaccination rollout programs in India [49]). For Russia, the projected rise in ASMR of cervical cancer attributable to both smoking and unsafe sex may reflect the persistently high prevalence of female smoking [50], the limited effectiveness of tobacco control policies [51], and suboptimal screening coverage coupled with substantial regional disparities in access to preventive care [52]. It should also be noted that some factors not included in our projection model like HPV vaccination schemes, screening programs, and HIV burden especially in some Low or Low-middle SDI countries, may further influence future disease burden, representing important considerations for interpreting long-term projections.

There were some advantages in our study. Firstly, this study is a large and systematic global analysis describing the epidemiological characteristics and shifts in the attributable burden of two risk factors for cervical cancer across different SDI levels of the world. Secondly, existing studies have focused on the descriptive analysis of disease burden for cervical cancer, while this study further explored the relationship between attributable burden of cervical cancer and SDI by using more comprehensive statistical methods such as quantile regression and restricted cubic spline. Thirdly, we predicted the mortality trends of cervical cancer caused by smoking and unsafe sex respectively, providing a scientific basis for future disease prevention and control of cervical cancer.

This study also had several limitations. Firstly, although we selected the most populous country within each SDI quintile to represent its respective development level, this approach cannot fully account for the heterogeneity that exists among countries within the same SDI category. Nevertheless, choosing population-dominant countries provides a reasonable approximation of the overall trends within each SDI level. Future studies could include multiple countries within each SDI group to more comprehensively capture regional variability in cervical cancer burden. Secondly, ecological fallacy is inevitable. This study is based on population-level observations and analysis, and individual-level associations cannot be confirmed. Thirdly, this study has not reported the burden of disease for different case types of cervical cancer. Lastly, our analysis relied on the GBD’s definition of “unsafe sex”, which is a broad metric that may not fully capture the nuances of individual-level risk in the modern context. For instance, it does not account for the risk-reduction impact of biomedical interventions such as Pre-Exposure Prophylaxis (PrEP) or the concept of Undetectable = Untransmittable (U = U) for HIV, which have transformed the landscape of sexual health.

This study also offers several key policy-relevant insights: First, we underscored smoking and unsafe sex as critical modifiable risk factors for cervical cancer, calling for integrated tobacco control and sexual health education—particularly in low-SDI regions where the burden is highest. Second, age-specific and regional disparities highlight the need for precision public health strategies, such as enhanced screening for women aged 55–59 and optimized care for elderly populations. Specifically, maybe it is necessary to add a further screening test at age 55–60 to screening all screening programs. Third, the significant and persistent inequalities across SDI levels underline the urgency of global cooperation and tailored support to low and low-middle SDI countries, providing a benchmark for monitoring progress toward cervical cancer elimination.

## 5. Conclusion

The global burden of cervical cancer is on the decline, but the situation remains serious. The primary burden was mainly from the loss of years of life due to premature death. Unsafe sex is the main risk factor for cervical cancer, followed by smoking. Targeted strategies should focus on distinct age groups: adults aged 55–59 years with highest incidence require enhanced prevention and screening, while those aged 95 years and older with highest mortality need optimized clinical care to reduce premature death. Low SDI and middle-low SDI areas are the key prevention and control areas for cervical cancer. Intervention of smoking and unsafe sex should be implemented in coordination with the major strategies (vaccination, screening, combined with HIV antiviral treatment when indicated) in the prevention of cervical cancer. Given the projected rising burden of smoking-attributable cervical cancer in China and Russian Federation, as well as unsafe sex-attributable cervical cancer in India and Russian Federation, integrating tobacco control or measures like sexual health education into cervical cancer prevention programs is particularly critical in these regions.

## Supporting information

S1 Fig The attributable ASDR of cervical cancer at different SDI quantile from 1990 to 2019. (**A)** The ASDR of cervical cancer attributable to smoking at different SDI quantile from 1990 to 2019. **(B):** The ASDR of cervical cancer attributable to unsafe sex at different SDI quantile from 1990 to 2019. DALY: disability-adjusted life-year; ASDR: age-standardized DALY rate; SDI: sociodemographic index.
(TIFF)

S2 Fig Age-specific mortality rates for cervical cancer attributable to smoking by SDI, 1990–2019. (TIFF)

S3 Fig The age-specific incidence rates for cervical cancer in the global and five different SDI quantiles, 1990 and 2019. (TIFF)

S4 Fig Trends in the proportion of YLLs for cervical cancer attributable to unsafe sex and smoking from 1990 to 2019. YLL: Year of life lost.
(TIFF)

S5 Fig The relationship between the ASDR (per 100000) of cervical cancer attributable to smoking, unsafe sex and SDI from 1990 to 2019 in 21 regions classified by GBD. Each colored line represents the time trend of the designated area. Each point represents a specific year for that region. The dotted line represents the result of quantile regression; from top down to next are the results of P_95_, P_75_, P_50_, P_25_ and P_5_. DALY: disability-adjusted life-year; ASDR: age-standardized DALY rate.
(TIFF)
