# Supplementary figures and images for: Global burden and projections of cervical cancer attributable to unsafe sex and smoking, 1990–2034
Source: PLoS One. 2026 Jan 9;21(1):e0339923. doi: 10.1371/journal.pone.0339923 (PMC12788690; doi:10.1371/journal.pone.0339923)

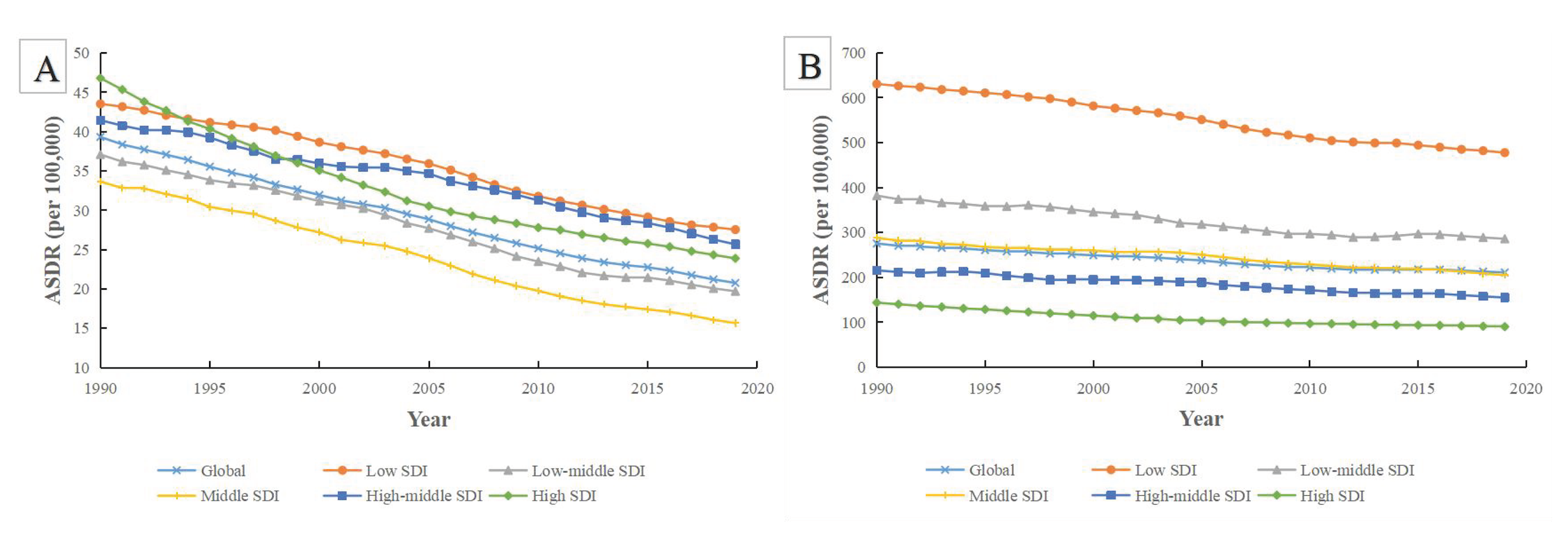

Supplement: S1 Fig — (A) The ASDR of cervical cancer attributable to smoking at different SDI quantile from 1990 to 2019. (B): The ASDR of cervical cancer attributable to unsafe sex at different SDI quantile from 1990 to 2019. DALY: disability-adjusted life-year; ASDR: age-standardized DALY rate; SDI: sociodemographic index. (TIFF) [file pone.0339923.s001.tiff]

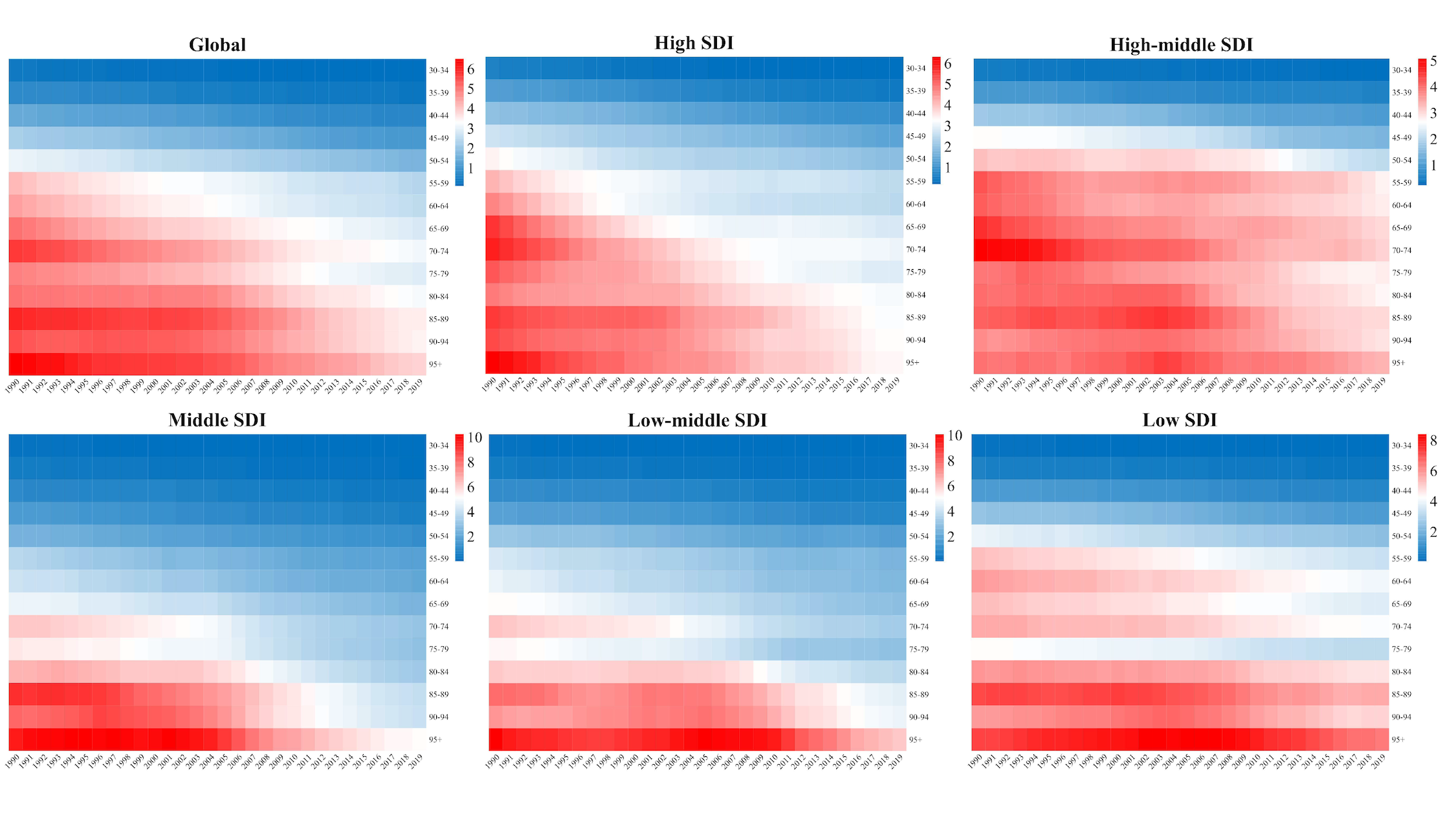

Supplement: S2 Fig — (TIFF) [file pone.0339923.s002.tiff]

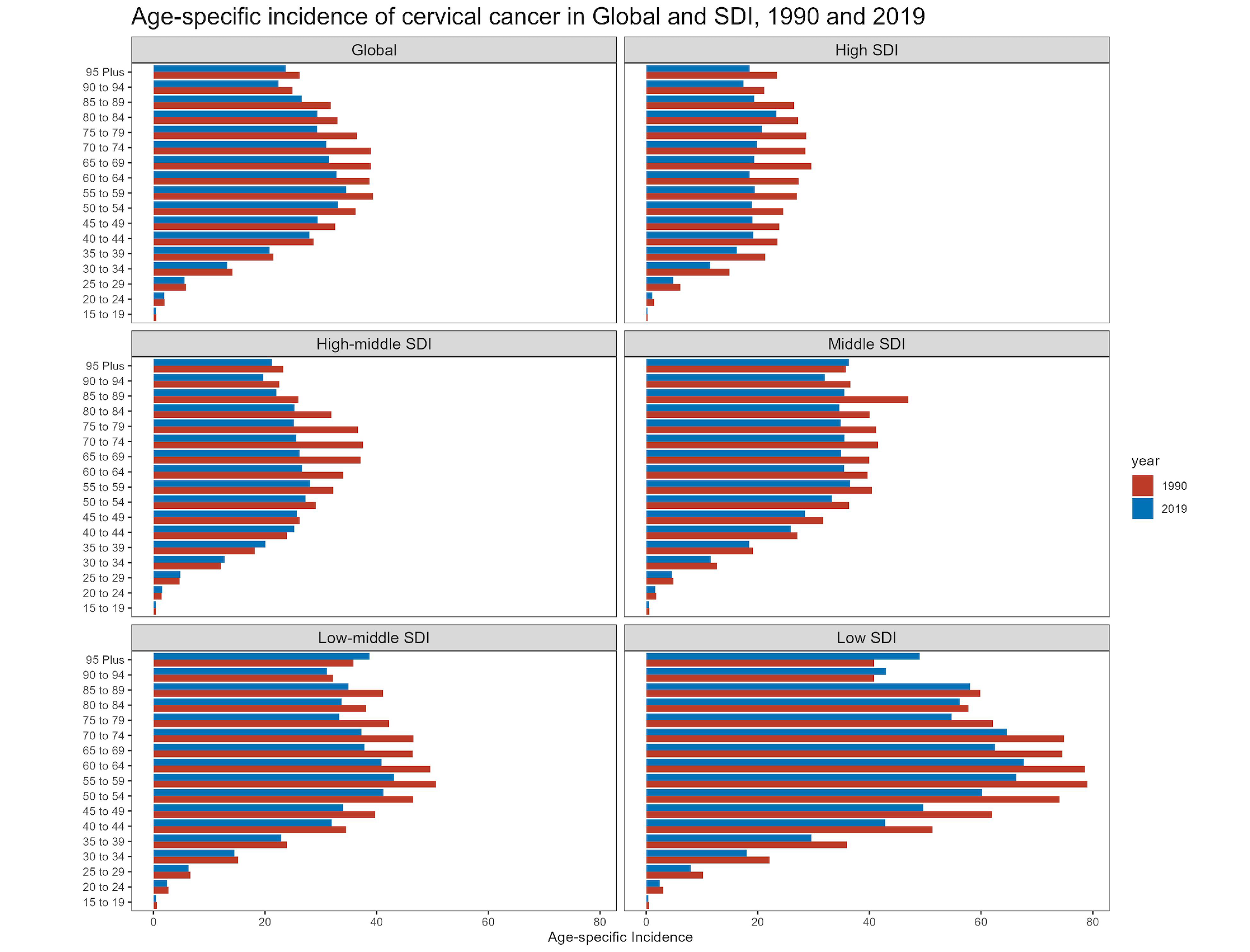

Supplement: S3 Fig — (TIFF) [file pone.0339923.s003.tiff]

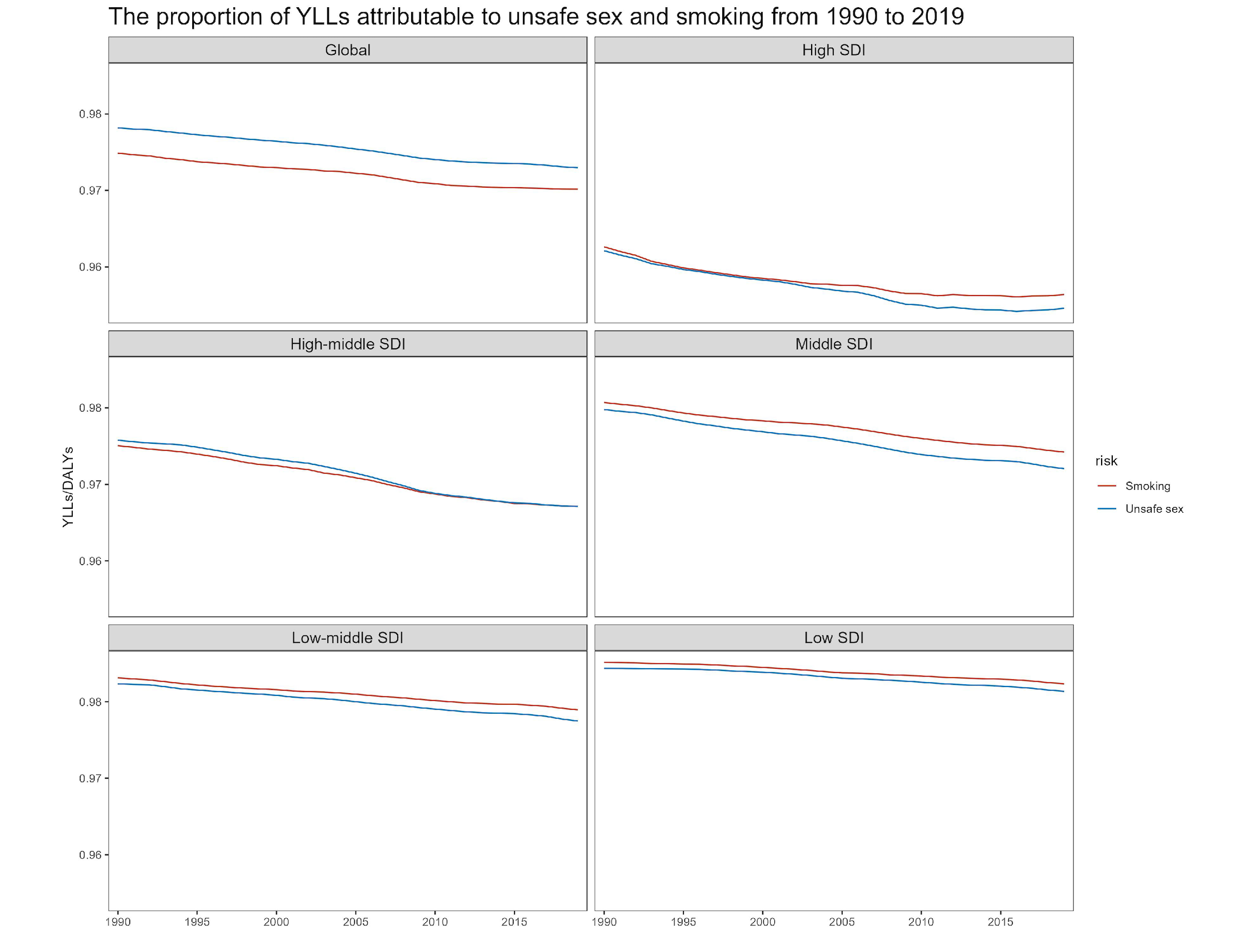

Supplement: S4 Fig — YLL: Year of life lost. (TIFF) [file pone.0339923.s004.tiff]

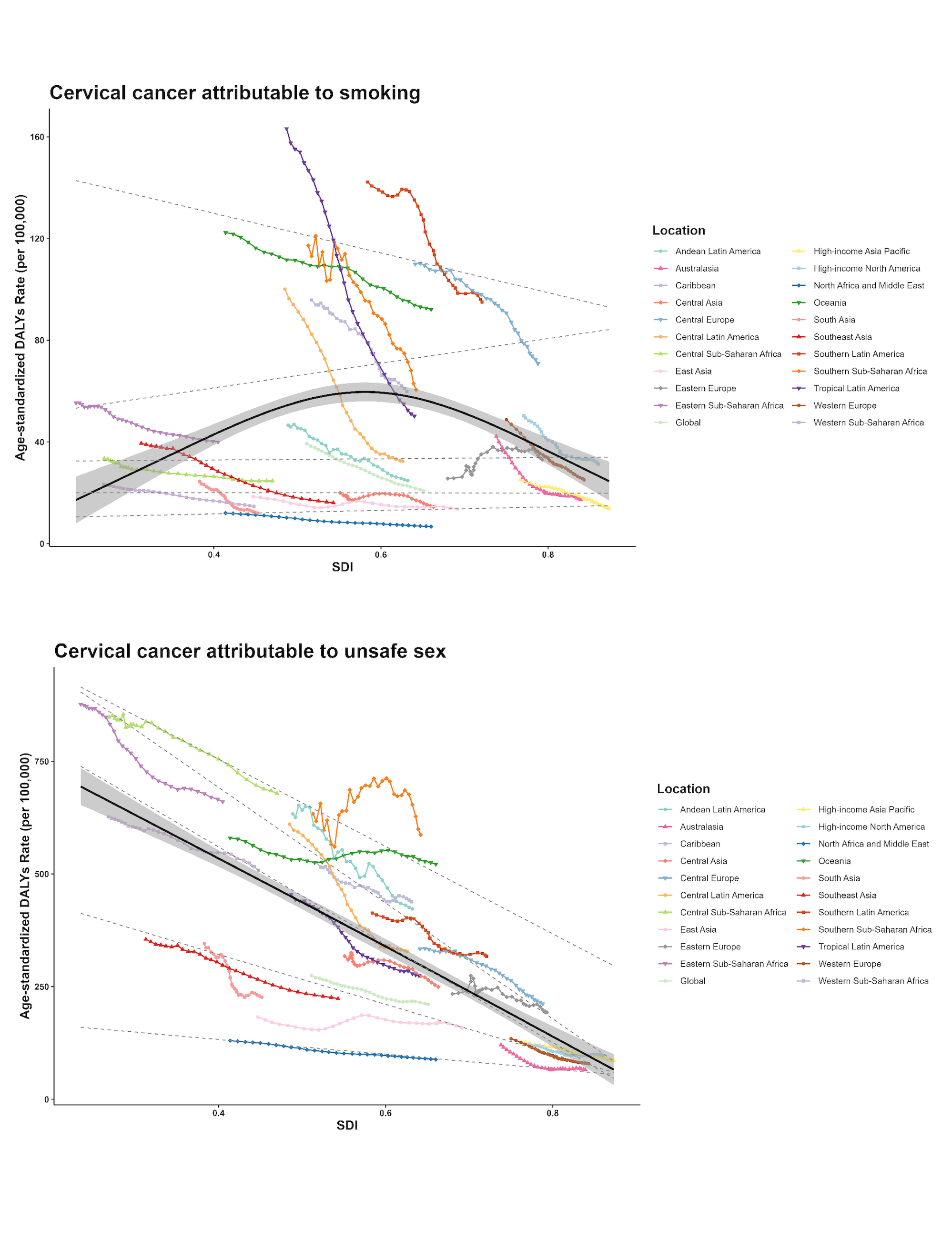

Supplement: S5 Fig — Each colored line represents the time trend of the designated area. Each point represents a specific year for that region. The dotted line represents the result of quantile regression; from top down to next are the results of P95, P75, P50, P25 and P5. DALY: disability-adjusted life-year; ASDR: age-standardized DALY rate. (TIFF) [file pone.0339923.s005.tiff]
